# *L. plantarum* UBLP-40 Versus the Combined Formula of *L. rhamnosus* UBLP-58 and *B. longum* UBBL-64 in Excisional Wound Healing: A Cellular Perspective

**DOI:** 10.3390/ph17111414

**Published:** 2024-10-22

**Authors:** Moysis Moysidis, Angeliki Chorti, Angeliki Cheva, Ioanna Abba Deka, Georgios Tzikos, Christoforos Kosmidis, Ioannis Koutelidakis, Joulia K. Tsetis, Theodossis Papavramidis, Katerina Kotzampassi

**Affiliations:** 1 Department of Surgery, Aristotle University of Thessaloniki, 54636 Thessaloniki, Greece; achorti@auth.gr (A.C.); tpapavra@auth.gr (T.P.); 2 Department of Pathology, Medical School, Faculty of Health Sciences, Aristotle University of Thessaloniki, 54124 Thessaloniki, Greeceampou_nt@hotmail.com (I.A.D.); 3 3th Department of Surgery, AHEPA University Hospital, Medical School, Faculty of Health Sciences, Aristotle University of Thessaloniki, 54124 Thessaloniki, Greece; 4 2nd Department of Surgery, G. Gennimatas University Hospital, Medical School, Faculty of Health Sciences, Aristotle University of Thessaloniki, 54124 Thessaloniki, Greece; 5 Uni-Pharma S.A., 14564 Athens, Greece

**Keywords:** probiotics, wound healing, *L. plantarum*, *L. rhamnosum*, *B. longum*

## Abstract

Introduction: The utilization of probiotics in enhancing the active healing of skin wounds represents a burgeoning trend in contemporary medicine. Previous research has extensively explored wound healing mechanisms involving the strains of *Lactiplantibacillus plantarum*, *Lacticaseibacillus rhamnosus,* and *Bifidobacterium longum*. This study seeks to compare and interpret cellular findings derived from immunohistochemical and pathological applications. Methods: Three groups (the control, *Lactiplantibacillus plantarum* (RO1) group, and *Lacticaseibacillus rhamnosus* and *Bifidobacterium longum* (PRO2) group) underwent histological analysis, and microscopic cell counting were employed, offering insights into dynamic changes among neutrophils, lymphocytes, plasmacytes, mast cells, fibroblasts, and newly formed vessels across distinct treatment groups and temporal intervals. Results: The neutrophil count was found to be elevated in PRO2 on day 2, while the same group resulted in the highest decline on day 15. The number of fibroblasts peaked on day 4 for the PRO1 group, compared to the other two groups, which peaked on day 8. The lymphocyte count was the highest in the control group, while they peaked on day 4 in PRO2. The mast cells and plasmacytes were variable and sparse among all groups and time frames. Neovascularization was promoted by PRO1 and PRO2 groups on day 4 and remained high on day 8 for PRO2. Conclusions: Probiotic strains can be beneficial to the human population and in assisting skin wound healing, each strain working differently and more effectively in different healing phases. Thus, a combined formula containing different probiotics to modulate various healing phases is desirable.

## 1. Introduction

Wound healing is a complex, curative response to tissue damage, leading to the restoration of tissue continuity, in an overlapping consequence of three phases: Initially, the blood clot plugs the bottom of the wound and acts as a temporary barrier to external pathogen invasion and debris. In parallel, coagulation factors indirectly promote neutrophil and macrophage migration to initiate the inflammatory phase. Subsequently, macrophages develop into M2 anti-inflammatory macrophages to guide tissue regeneration in alignment with fibroblast migration. As a result, the granulation tissue consisting of macrophages, fibroblasts, connective tissue, and new blood vessels develops to replace the initial blood clot. Furthermore, fibroblasts differentiate into α-SMA-expressing myofibroblasts to increase the wound stiffness/tension. Finally, wound cellularity decreases, and collagen type III in the extracellular matrix is replaced by collagen type I to form the scar tissue [1,2].

Skin homeostasis is regulated by microorganisms, known as the skin microbiota, which influence keratinocytes and their cytokine release, thereby maintaining optimal skin condition [3]. These microorganisms typically serve as the first line of defense by producing antimicrobial peptides and enhancing the immune capabilities of toll-like receptors, Langerhans cells, and T cells [4]. After injury, the skin not only experiences evident loss of integrity but also demonstrates changes in the beta-diversity of its resident microbiota [5]. Such disruptions in the local microbiota may trigger a sequence of significant processes, including dysregulation of the immune and inflammatory response, delayed re-epithelialization, and fibrin deposition at the cellular and molecular levels [5,6,7].

According to the current definition endorsed by both the Food and Agriculture Organization of the United Nations (FAO) and the World Health Organization (WHO), as well as the International Scientific Association for Probiotics and Prebiotics (ISAPP), “probiotics are live microorganisms that, when administered in adequate amounts, confer a health effect on the host” [8]. Probiotics exert potential suppressive effects on various infectious, immune-related, and inflammatory conditions. Several animal studies have suggested that a probiotic regimen can be beneficial for tissue repair, decrease bacterial load, and promote the development of a microbiome that may positively influence the structural components of the skin [9,10,11]. Since different probiotic strains exert varied anti-inflammatory responses, it is speculated that they are mainly involved in the early inflammatory phase of healing, as well as during the stages of fibroblast differentiation and collagen production [12,13,14].

Various experimental and clinical investigations have substantiated the role of probiotics in the healing process through diverse mechanisms. These include facilitating the inflammatory phase similar to an anti-inflammatory agent and fostering epithelialization by stimulating fibroblast and collagen mobilization. Given the unique mechanisms through which each probiotic strain facilitates the healing process, and knowing the potential collaboration of different probiotic strains to enhance their efficacy, it is pertinent to delve into the cellular intricacies of these differences in the healing process.

In a recent work of our team, we found that the topical application on a full-thickness excisional wound model in rats of either a single probiotic strain, the *L. plantarum* UBLP-40, or the commercially available combo formula of *Lacticaseibacillus rhamnosus* UBLP-58 and *Bifidobacterium longum* UBBL-64, significantly improved the healing process versus control, but each treatment formula worked differently in each phase of the healing process [15]. Thus, the objective of the current study is to compare step-by-step the healing properties of these two formulas by means of histomorphology. This is accomplished by focusing on the cellular changes in the wound bed at each stage of wound healing. More specifically, the numbers of neutrophils, fibroblasts, lymphocytes, mast cells, and plasmacytes are counted on days 0, 2, 4, 8, and 15 of the injury. We also calculate the neovascularization of the wound at each time point.

## 2. Results

### 2.1. Neutrophils

The first (inflammatory) phase of healing (from day 0 to 4)begins with neutrophil migration toward the wound area. In the control (no treatment) group, neutrophils exhibited a sudden increase on day 2 (median value 41, interquartile range (iqr): 52), followed by a decline in their number to almost one-third on day 4, and then progressively disappeared (days 8 and 15). At the corresponding time phases, the PRO1 group presented a very limited increase in neutrophils (median 20.5, iqr: 20), significantly smaller than that of the control group (*p*: 0.036), and then a further decline.

**Figure 1:**
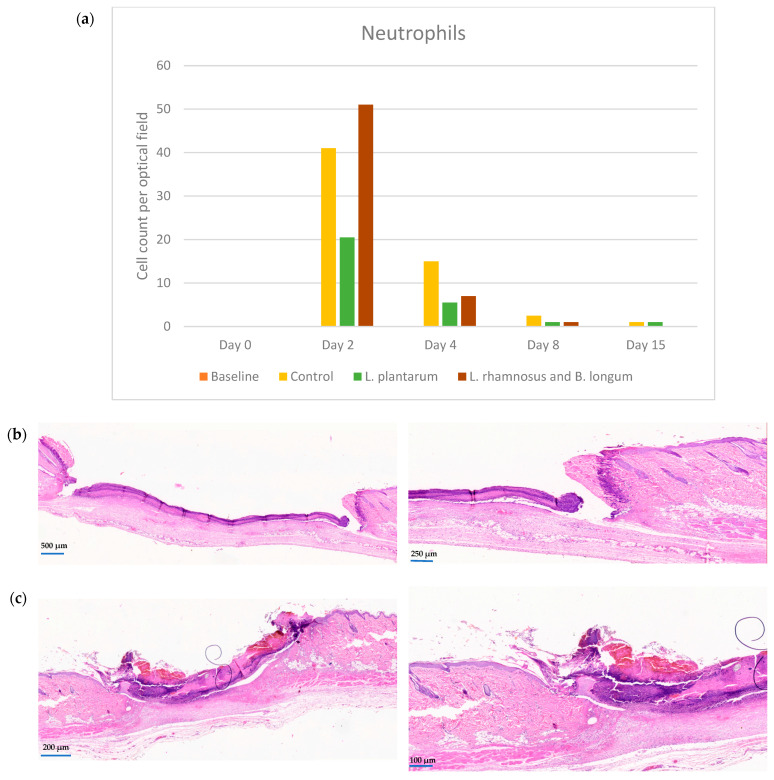

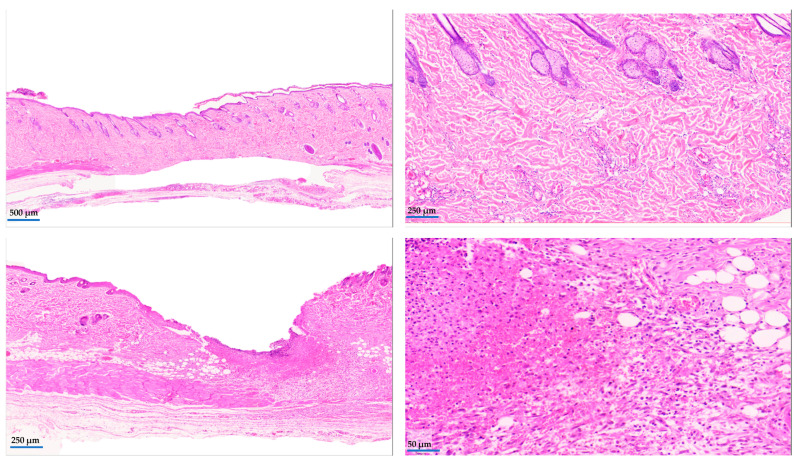
(**a**) Neutrophil counts among the control, *L. plantarum* (PRO1) and *L. rhamnosus* and *B. longum* (PRO2) groups on days 2, 4, 8, and 15. The day intervals are represented on the *x*-axis, while the number of neutrophils is depicted on the *y*-axis. (**b**) Histological images of the control group on day 2, ×10 and ×40. (**c**) Histological images on day 4. First row: control group at ×10 and ×40; second row: *L. plantarum* (PRO1) group at ×10 and ×40; third row: *L. rhamnosus* and *B. longum* (PRO2) group at ×10 and ×40. The neutrophil count was significantly increased in the PRO2 group (double regimen).

Conversely, the PRO2 group presented the highest number of neutrophils on day 2 (median 51, iqr: 77), higher than both the control’s and the PRO1 group on the same day (*p*: 0.787 and *p*: 0.001, respectively), followed by a rapid drop of their number on day 4 (median 7 iqr: 10); the value was significantly lower than the control (median 15, iqr: 14) but higher than *L. plantarum* (PRO1 group) (median 5.5, iqr: 7) (*p* < 0.001). In other words, *L. plantarum* (PRO1 group), known for its anti-inflammatory properties, seems to “prohibit” the excess migration of neutrophils towards the wounded area, thus keeping the local inflammation to a minimum in relation to both the control and the double regimen (PRO2 group), which exert the highest migration (Figure 1a–c).

### 2.2. Fibroblasts

In the control group, fibroblasts, attracted by mediators, were found to significantly increase in number, beginning from day 2, reaching a peak (almost 6-fold) on day 8, and then decreasing until day 15. The number of fibroblasts in the PRO2 group also increased significantly at every phase, paralleling the controls but with significantly lower values compared to the control group. During neovascularization (day 4), a significantly elevated number of fibroblasts in the double regimen group was observed, almost three times higher on day 8, with a median value of 64 (iqr: 62). A similar trend was observed in the control group, with the median fibroblast count increasing from 45.5 (iqr: 38) on day 4 to 119 (iqr: 52) on day 8 (*p* < 0.001).

**Figure 2:**
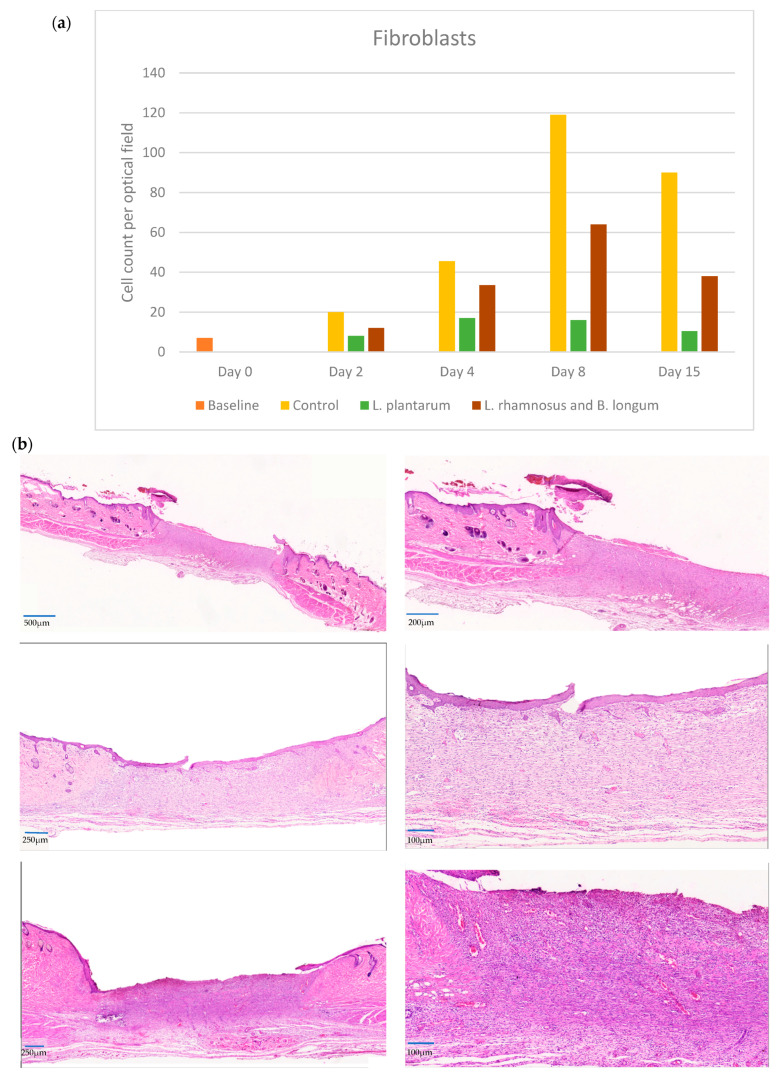
(**a**) Fibroblast counts among the control, *L. plantarum* (PRO1) and *L. rhamnosus* and *B. longum* (PRO2) groups on days 2, 4, 8, and 15. The day intervals are represented on the *x*-axis, while the number of fibroblasts is depicted on the *y*-axis. (**b**) Histological images on day 8. First row: control group at ×10 and ×40; second row: *L. plantarum* (PRO1) group at ×10 and ×40; third row: *L. rhamnosus* and *B. longum* (PRO2) group at ×10 and ×40. The overall number of fibroblasts was increased in the PRO2 group.

However, in the *L. plantarum* group, the peak occurred earlier on day 4, reaching a median value of 17 (iqr: 6), which was significantly lower than in the other groups and remained lower until day 15 (*p* ≤ 0.001). Τhe *L. plantarum* (PRO1) group exhibited a significantly lower number of fibroblasts throughout the study phases, with a small peak on day 4, followed by a decline. This finding, similar to what can be observed with the neutrophil count, underlines the strong anti-inflammatory properties of *L. plantarum*, which also is known for its anti-fibrotic action (Figure 2a,b).

### 2.3. Lymphocytes

In control-treated wounds, lymphocytes presented a significantly high value on day 2 that progressively decreased over the next days/phases. They were present in a similar way in the PRO1 group, but their numbers were consistently less than half of those in the control group during each phase of the study. The control and the *L. plantarum* groups both followed a similar downward trend in the number of lymphocytes. On day 2, the median number of lymphocytes in the control group, reaching 37 (iqr: 45), was significantly higher than the *L. plantarum* group’s count of 15.5 (iqr: 19, *p* < 0.001). By day 15, the *L. plantarum* group displayed the lowest median number of lymphocytes, measuring 4 (iqr: 2), compared to the control group (12, iqr: 14) and the double regimen group (PRO2, median 14.5, iqr: 15) (*p* < 0.001).

**Figure 3:**
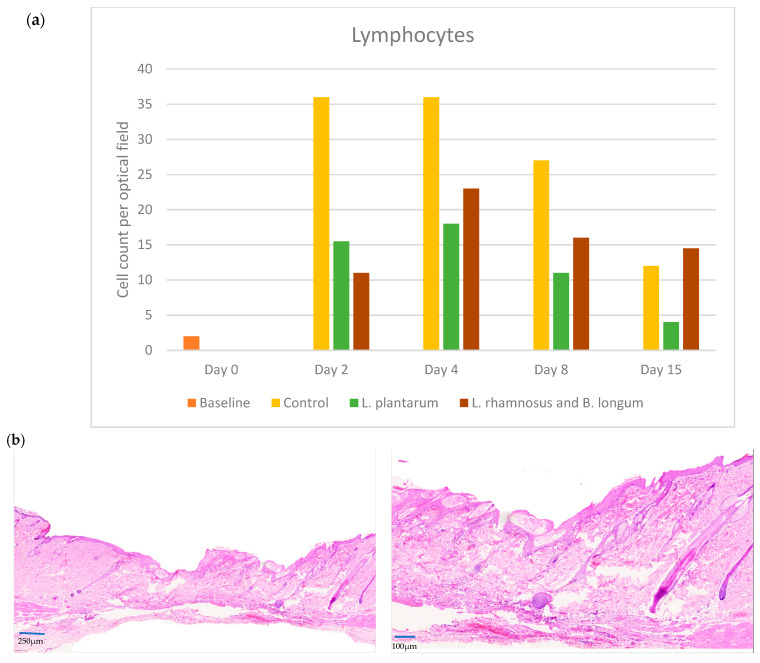

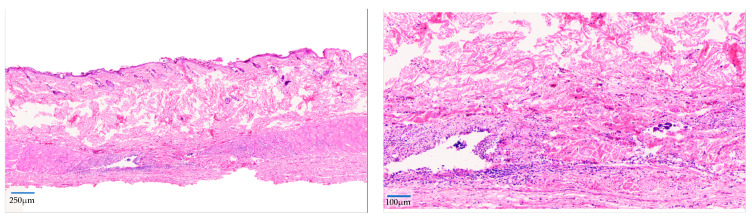
(**a**) Lymphocyte counts among the control, *L. plantarum* (PRO1), and *L. rhamnosus* and *B. longum* (PRO2) groups on days 2, 4, 8, and 15. The day intervals are represented on the *x*-axis, while the number of lymphocytes is depicted on the *y*-axis. (**b**) Histological images on day 4. First row: PRO2 group at ×10 and ×40; second row: control group at ×10 and ×40. The control group had the highest number of lymphocytes on day 4.

Conversely, in the *L. rhamnosus* and *B. longum* (PRO2) group, lymphocytes progressively increased from day 2, peaking on day 4, and then decreased. On day 2, lymphocyte count was the lowest among all groups. There is a peak in the median number of the lymphocytes, reaching 23 (iqr: 17) during the proliferative phase (day 4), while, on day 15, there was a decrease in the mean lymphocyte number, which remained higher than the other groups’ counts on the same day. However, although their number is generally smaller than in the control group and higher than those in the *L. plantarum* PRO1 group, they remained high on day 15, when the numbers in the other groups had decreased (Figure 3a,b).

### 2.4. Mast Cells

**Figure 4:**
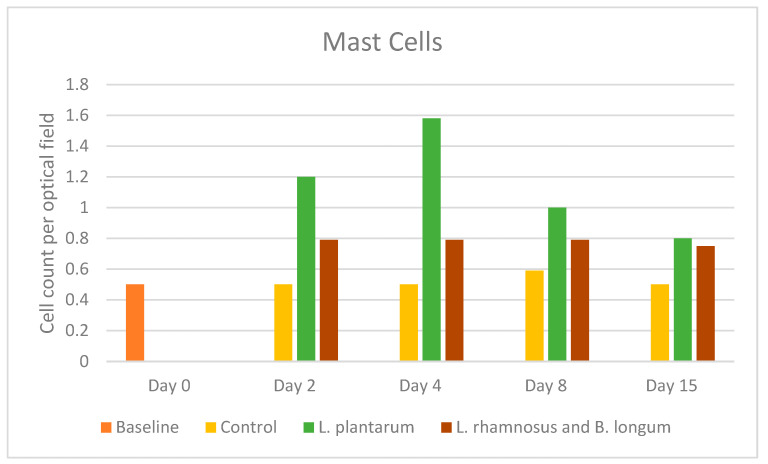
Mast cell counts among the control, *L. plantarum* (PRO1), and *L. rhamnosus* and *B. longum* (PRO2) groups on days 2, 4, 8, and 15. The day intervals are represented on the *x*-axis, and the number of mast cells is shown on the *y*-axis.

In control-treated wounds, mast cell counts were almost similar in all the healing phases, with a non-significant increase only on day 8, with a mean value of 0.59 (SD: 0.59). The same was observed in the PRO2 group, with no fluctuations in the cell number, although their numbers were consistently higher compared to the control group at each time point. Τhere is an increase in mast cell count on day 2, and then a non-diverging mean value of 0.79 is observed (D2 SD: 1.25, D4 SD: 0.98, D8 SD: 1.29). In contrast, the PRO1 group presented significantly higher values at all time points, beginning on day 2, peaking on day 4 (mean value of 1.58 (SD: 1.77)), and then decreasing to match the value of the PRO2 group on day 15 (Figure 4). Νo statistically significant comparison was observed among study groups.

### 2.5. Plasmacytes

**Figure 5:**
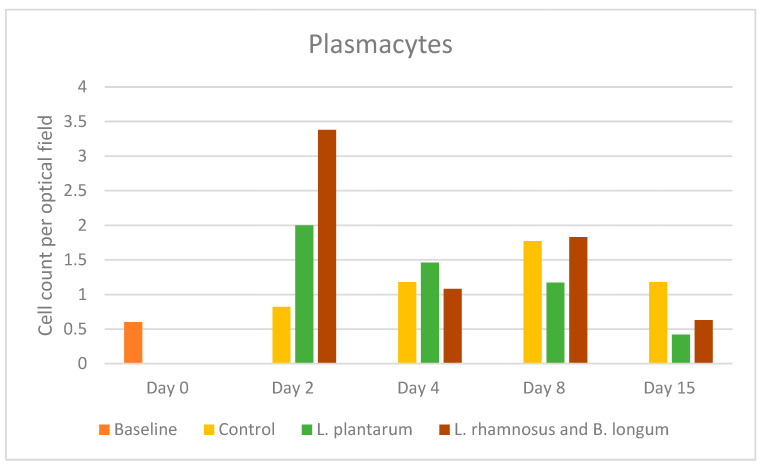
Plasmacyte counts among the control, *L. plantarum* (PRO1), and *L. rhamnosus* and *B. longum* (PRO2) groups on days 2, 4, 8, and 15. The day intervals are represented on the *x*-axis, and the number of plasmacytes is shown on the *y*-axis.

In control-treated wounds, plasmacytes were present in low numbers, peaking on day 8 with a mean value of 1.77 (SD: 1.63). In the *L. plantarum* (PRO1) group, a significant increase—more than double the control’s number—was found on day 2, followed by a progressive decrease to a minimum by day 15. In the *L. rhamnosus* and *B. longum* (PRO2) group, a highly significant increase—more than threefold the control’s number—was found on day 2 (its highest mean value of 3.38 (SD: 4.2)), followed by a sudden drop on day 4, reaching the second lowest mean value (1.08 (SD: 1.84)), and a new increase on day 8, to reach half the number of cells found in the control group, on day 15, measuring 1.83 (SD: 3.54) and 0.63 (SD: 0.65), respectively. No statistically significant values were noted (Figure 5).

### 2.6. Angiogenesis Is Enhanced by L. plantarum Group on Days 4 and 8

**Figure 6:**
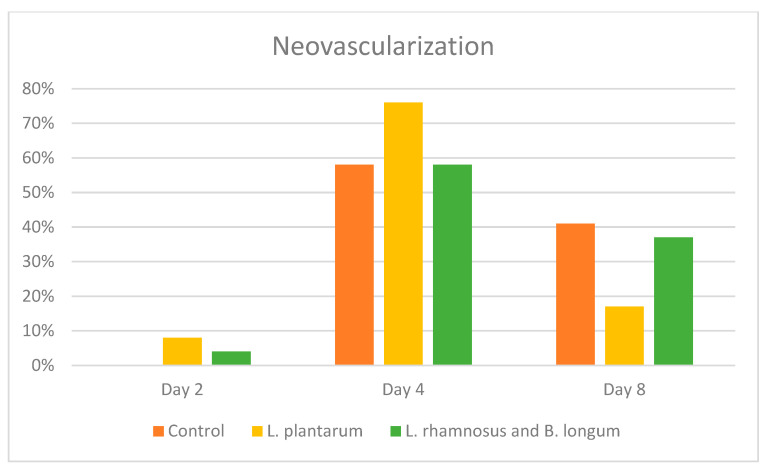
Neovascularization among the control, *L. plantarum* (PRO1), and *L. rhamnosus* and *B. longum* (PRO2) groups on days 2, 4, and 8.

The presence of angiogenesis in the depth of the ulcer ranged from 1 to 39%, defining a positive result in the immunohistochemical analysis as a tissue reaction > 5%. Based on this speculation, the PRO1 and PRO2 groups appeared to enhance angiogenesis from day 4 to day 8 compared to the control group. Between the two groups of probiotic regimens, PRO1 was found to promote the development of new vessels in the wound area more on days 4 and 8, compared to the PRO2 group, which scored lower. However, the PRO2 group maintained its efficacy on neovascularization on day 8, at a higher rate than the PRO1 group (Figure 6).

## 3. Discussion

Excisional wounds, whether involving smaller or larger skin loss, remain a troublesome postoperative issue. The aim is a quick and prompt restoration of the skin barrier function in order to prevent further damage or infection and to achieve an aesthetically acceptable scar formation.

Probiotics have proved to promote wound healing by inhibiting the growth of pathogenic bacteria, regulating local inflammatory response, and interacting with epidermis cells [16]. *L. plantarum* exerts a stronger anti-inflammatory effect compared to the combination of *L. rhamnosus* and *B. longum* regimens, while the double regimen enhances angiogenesis and the expression of healing factors [17]. This study focuses specifically on the changes in the cellular populations involving the *Lactiplantibacillus plantarum* and the combined regimen of *Lacticaseibacillus rhamnosus* and *Bifidobacterium longum* strains.

The studied procedure of wound healing influenced by *L. plantarum* and the double regimen began on day 2, during the inflammatory phase, which marks the initial response to tissue injury. The tissue de-cohesion causes blood vessel disruption and extravasation of blood constituents. The wound is characterized by a hypoxic environment in which a fibrin clot, as well as the neighboring damaged tissue cells, facilitate the secretion of vasoactive mediators and chemotactic factors. The first responders of the immune system, the neutrophils, rapidly migrate to the wound site. There, they play a crucial role in phagocytizing debris, pathogens, and dead and devitalized cells, as well as releasing reactive oxygen species and proteolytic enzymes contributing to the breakdown of damaged tissue. These neutrophils express genes that are different from circulating neutrophils [18]. It is noteworthy that neutrophils did not appear in the neighboring healthy skin nor in the samples taken on day 0 in our study.

Regarding neutrophils among the three predetermined groups, the *L. plantarum* group showed decreased numbers of neutrophils throughout the duration of our study, supporting its anti-inflammatory response. The double regimen group showed an elevation in neutrophil count, which could be attributed to the pro-inflammatory effect of the *Lacticaseibacillus rhamnosus* and *Bifidobacterium longum* combination, as supported by earlier publications [17]. Neutrophils exhibit a remarkably short lifespan, undergoing apoptosis within hours of their recruitment, and later phagocytosed by macrophages, preventing an uncontrollable inflammatory response [19]. It is important to note that the mean number of neutrophils in the *L. plantarum* group is significantly lower compared to the other two groups, resulting in a shorter duration of the inflammatory response. This could be a cofactor for the rapid reepithelization seen by day 4 in this group. In the study by Nasrabadi et al., applying *L. plantarum*, the neutrophil count increased on day 3 and then decreased by day 5, indicating the pivotal role of *L. plantarum* in wound healing by stimulating the immune system in the early phase and dampening it in the late phase [20]. *L. plantarum* has been recognized as a strong anti-inflammatory agent that promotes neutrophil and macrophage attraction [17,21,22,23]. Regarding *B. longum*, in an ex vivo study, a significant improvement was observed in parameters relating to inflammation, such as a decrease in vasodilation, oedema, mast cell degranulation, and TNF-alpha release compared to placebo [12].

Epidermal T cells are sentinel and sparse in the epidermis and are among the first responders after tissue injury. Regarding lymphocyte analyses, the control group displayed the highest numbers of lymphocytes on day 2, whereas in the double regimen group, the lymphocyte number peaked on day 4, then dropped but remained elevated in comparison to the other groups. In the *L. plantarum* group, the lymphocyte count remained lower on days 4 to 15 compared to the control group. During wound healing, keratinocytes express a currently unknown antigen that activates dendritic T-cell-activated lymphocytes in cell cultures. In in vivo studies, the response of epidermal T cells is rapid, within hours of tissue damage [24]. Also, within 48 hours after injury, cutaneous T cells generate factors that increase the number of keratinocytes at the wound site. It is noteworthy that agents that suppress T lymphocyte function substantially impair the wound healing mechanism [25]. *L. plantarum* was found to induce keratinocyte migration, resulting in earlier wound re-epithelization on day 12, compared to the control group on day 15 [22]. B cells have been linked to angiogenesis, neuroregeneration, and collagen deposition [26]. In terms of plasmacytes, the trend in probiotic regimens is notable. In the *L. plantarum* group, plasmacyte count peaked on day 2 and then dropped gradually, while in the double regimen group, they peaked once on day 2, reaching the highest values compared to the other groups, and then again on day 8, when their levels were similar to the control group. This result aligns with other studies showing infiltration of the wound bed by activated B cells in low numbers at 3 and 7 days after injury [27]. Plasma cell contribution to wound healing is hypothesized to involve the opsonization of dead cells by producing IgG1 immunoglobin. They can also indirectly assist in the reduction of inflammation by affecting the expression of cytokines through the TGF-β pathway [26].

Upon tissue injury, mast cells are activated and initiate a secretory response that includes the release of an array of cytoplasmic granule-associated mediators, promoting vasodilation and vascular permeability, thus significantly promoting the healing cascade. Mast cells also promote fibroblast collagen synthesis and deposition. It should be noted that in murine studies, mast cells have been closely associated with microbial skin resistance and nematode resistance [28,29]. As the inflammatory phase progresses, specifically after two to three days, monocytes infiltrate the injured tissue and further differentiate into macrophages. Pro-inflammatory macrophages contribute to further phagocytosis of debris and apoptotic cells, while releasing pro-inflammatory cytokines such as interleukins (IL-1, IL-6, IL-8, IL-12), transforming growth factor A (TFG-α), tumor necrosis factor-alpha (TNF-α) in response to bacterial products, as well as angiogenic factors [30,31]. The number of mast cells in our study in the *L. plantarum* group increased on day 2 and peaked on day 4, while in the double regimen group, they peaked on day 2 and remained stable until day 15. Both probiotic groups showed higher values of mast cells compared to the control group throughout the wound-healing process.

During the late inflammatory phase of wound healing, fibroblasts are mobilized toward the wound site, due to the presence of chemokines and growth factors produced by neutrophils and macrophages. Once positioned, fibroblasts interact with the surrounding immune cells, releasing factors that promote the immune response by enhancing phagocytosis [32]. Nasrabadi et al. proposed *L. plantarum* as the main contributor in this healing phase, inducing macrophage and fibroblast increases on day 7, initiating the collagen deposition phase earlier [20]. An earlier increase in fibroblasts was also seen in two other studies in the *L. plantarum* group compared to the control group, suggesting a significant reduction in local inflammation [33,34].

The transition to the proliferative phase is marked by a shift in cellular activities toward tissue repair and regeneration. The second stage of tissue repair, called reepithelization, occurs two to ten days after the initial injury. The re-epithelialization process commences as the wound’s inflammatory phase subsides. Various cytokines, growth factors, and chemokines that guide the recruitment and activation of keratinocytes are involved. Transforming growth factor-alpha, epidermal growth factor (EGF), and keratinocyte growth factor (KGF) play pivotal roles, stimulating keratinocyte migration, proliferation, and differentiation [35]. Both treatments associated with the tested probiotic regimens, significantly boosted healing factor expression, with the double regimen group exhibiting a stronger impact on certain factors like TGF-β. This anti-inflammatory cytokine has been associated with the faster wound-healing process in the *L. plantarum* group [36]. In a study by Dubey et al., comparing the *L. plantarum* and control groups, histopathology revealed on day 7 an enhanced proliferation of fibroblasts, angiogenesis, re-epithelization, collagen deposition, and granulation tissue were observed in the *L. plantarum* group. Furthermore, the wounds were totally healed on day 14 in the *L. Plantarum* group, while incomplete healing was observed in the control group [23]. In addition, enhanced angiogenesis, reduced leukocyte infiltration, granulation tissue, and mast cell counts were observed by the application of *L. rhamnosus* on wound healing [37]. *L. rhamnosus* is also more effective in keratinocyte migration due to increased expression of CXCL2 chemokine and its receptor [16]. Furthermore, *L. plantarum* was found to accelerate re-epithelization through the promotion of keratinocyte migration [38].

Another event of the proliferative stage is angiogenesis, which is necessary to preserve the newly formed granulation tissue. This process ensures an adequate supply of oxygen and nutrients to support the healing process. Endothelial cells within existing blood vessels are activated by angiogenic factors like vascular endothelial growth factor A (VEGFA) and fibroblast growth factor 2 (FGF2) to migrate toward the wound site and form new capillaries [35]. Angiogenesis is also induced by neutrophils, meaning that, as *L. plantarum* enhances the earlier reduction of neutrophils, it promotes the earlier onset of angiogenesis [39,40,41]. Mice treated with *L. plantarum* showed increased angiogenesis on day 7 [23]. However, a study by Panagiotou et al. suggested that the *L. rhamnosus* and *B. longum* combination, but not *L. plantarum*, enhances angiogenesis [17]. In our study, we found that both probiotic regimens showcased enhanced neo-angiogenesis, which had also begun earlier than in the control group, while the *L. plantarum* group scored the highest percentage of newly formed vessels in the depth of the wound.

During the proliferative phase, there is marked fibroblast migration, replication, extracellular matrix synthesis, and deposition. Various factors, produced by platelets and other immune cells, stimulate fibroblast proliferation and promote fibroblast transition into a more synthetic phenotype [32]. This proliferative process is evident when examining the fibroblast number in the current study. It is also noted that in the *L. plantarum* group, the mean number of fibroblasts was significantly lower in comparison to the other two groups, supporting the fact that *L. plantarum* could prevent hypertrophic scar or keloid formation. This hypothesis is supported by the Panagiotou et al.’s study, suggesting that *L. plantarum* could reduce hypertrophic scar formation by attenuating healing factors [17]. *L. plantarum* exerts an earlier dose-dependent effect on a-SMA inhibition, which also supports the inhibitory action of *L. plantarum* on excessive skin fibrosis [42,43]. This further supports the crucial cellular event of neovascularization, which is necessary to preserve the newly formed granulation tissue. The mean number of fibroblast peaks by day 4 in the *L. plantarum* group instead of day 8, as in the control group and double regimen group. The fibroblasts, which can be sourced either from the edges of the wound or by the bone marrow, are further differentiated into myofibroblasts after maturation [44]. The mean number of fibroblasts in the *L. plantarum* group is significantly lower compared to the double regimen group, even though the proliferative response might be accelerated in comparison to the other two groups. This could be an explanation of the reversal in fibroblast numbers, with the double regimen group showing progressively more effective wound healing up to day 15.

Multiple efforts during the last decade have attempted wound visualization and configuration. These imaging methods may involve various applications including medical suture applications, photodynamic therapy, and probiotic applications [15]. For example, Li et al. introduced a novel technique for qualifying 3D wound closure in living animals as well as analyzing leukocyte populations from wound dermis and epidermis using flow cytometry. The application of this noninvasive method has proven necessary to examine wound healing defects and assist treatment [45].

Further publications have examined the skin configuration, comparing the single regimen of *L. plantarum* and a double regimen of *L. rhamnosus* plus *B. longum* [15]. The *L. plantarum* group initiates the healing process earlier than the *L. rhamnosus* plus *B. longum* group, with the wounded area reduced by 41.2% on day 4, as opposed to 12.2% in the control group and 29.5% in the double regimen group. The *L. plantarum* group also exhibits a significantly greater healing response than the double-regimen group on day 2, marking the proliferative phase, which is indicated by wound area reduction, S length, and D length measurements. Similar studies in diabetic wound healing in Wistar rats showed accelerated closure rates by day 7 in probiotic-treated wounds with *L. plantarum* compared to the control. In these studies, by day 14, *L. plantarum*-treated wounds showed over 95% closure, significantly higher than the control group (70 ± 9.6%). Also, probiotic-treated wounds showed fewer inflammatory cells by day 7 and increased fibroblast cells on days 7 and 14 compared to the control group [36]. Specific proteins induced by *L. plantarum* and *L. salivarius* were related to wound-healing properties and keratinocyte migration [39]. These data are in agreement with the results of the current study.

Various animal studies, including this one, have indicated the positive effects of probiotics in enhancing tissue repair as well as reducing bacterial load and producing a microbiome that can have favorable outcomes for the skin’s structural elements [9,10,11]. Topical probiotic regimens have shown efficacy in healthy patients by reducing inflammation, increasing ceramide concentration, and improving skin hydration [12].

Depending on the aim of the healing process that needs to be altered, strains such as *Lactiplantibacillus plantarum*, *Lacticaseibacillus rhamnosus*, and *Bifidobacterium longum* can be of help. The pro-inflammatory effect of the *Lacticaseibacillus rhamnosus* and *Bifidobacterium longum* combination could be beneficial for patients with neutropenia or other hematopoietic complications that alter the normal quality of neutrophil function. The *Lactiplantibacillus plantarum* regimen can be beneficial for patients with diabetic ulcers or other forms of vascular disease that create a hypoxic environment. In this study, as well as in older publications, the crucial cellular event of neovascularization, which can be altered by various factors, can be promoted by the double regimen group.

### Limitations

The present study has some limitations. Firstly, rat skin does not perfectly mimic human skin because rats are described as loose-skinned animals, allowing contraction to play a significant role in the approximation of the rat skin margins, while the presence of panniculus carnosus muscle, which does not exist in humans, contributes to final wound healing by both contraction and collagen formation. Furthermore, the double regimen group was composed of two strains, *L. rhamnosus* and *B. longum*. Ideally, we should also have tested each one of these strains separately. However, our purpose was to compare a single strain with a combination of strains; unfortunately, *L. rhamnosus* and *B. longum* were available from our donors only as a mixture.

## 4. Materials and Methods

### 4.1. Animals

Fifty-two healthy male Wistar rats weighing 200–250 g were individually housed in polypropylene cages under controlled environmental conditions for acclimatization. Water and food (standard laboratory chow diet) were provided ad libitum, and the rats were allowed to acclimatize for a week. Housing, anesthesia, wound induction, and postoperative care complied with the European Guidelines for the Care and Use of Laboratory Animals. The experimental protocol was approved by the Local Governmental Committee for the Control and Supervision of Experiments on Animals (EU Directive 2010/63/EU, protocol registration number 227933(934)/06.05.2021).

### 4.2. Study Design

After the acclimatization period, rats were randomly allocated into three groups, while another group of four rats served as the baseline measurement group (time 0), which were then immediately sacrificed. The three groups were as follows: the control group (the placebo-treated rats); the *L. plantarum* group (Lp group—rats treated with *L. plantarum*, PRO1); and the combo regime group (Combo group—rats treated with the combo regime of *Lacticaseibacillus rhamnosus* plus *Bifidobacterium longum*, PRO2). Each of the three study groups included 16 rats, four animals for each time point of the study, for the assessment of healing progress on the 2nd, 4th, 8th, and 15th days post-injury (Table 1).

### 4.3. Wound Induction

Rats were anesthetized intraperitoneally with ketamine (100 mg/kg) plus xylazine (10 mg/kg); the dorsal skin was shaved with an electrical clipper, rinsed with an alcohol swab, sterilely prepped with betadine, and finally draped with sterile sheets. Six full-thickness open excision wounds extending through the panniculus carnosus were surgically performed in the dorsal skin, using a sterile 8 mm diameter biopsy punch. A “blinded” operator filled the excisional wound area with the treatment formulae (see Section 4.4), according to the allocation group. Wounds were then immediately covered with sterile gauze dressing, reinforced with a self-adhesive bandage, and the rats were returned to their own individual cages to recover from anesthesia. Treatment was re-applied every two days, with the rats under ether anesthesia.

### 4.4. Probiotic Treatment

The probiotics used in this study were as follows: *Lactiplantibacillus plantarum* UBLP-40 (PRO1 group) and a combined formula of *Lacticaseibacillus rhamnosus* UBLR-58 plus *Bifidobacterium longum* UBBL-64 (PRO2 group). They were delivered as a fresh, purified stock culture in the form of a dry powder containing 10^11^ cfu/gr, donated by the Pharmaceutical Company UniPharma, SA, Athens, Greece. For use in this experiment, 0.3 mL of normal saline (0.9%) was freshly added to each pre-weighed gram of dry probiotic culture, making it a type of “ointment” ready for application to the wound. The same volume of 0.3 mL of normal saline (0.9%) was used as a placebo treatment in the corresponding group (Contr).

### 4.5. Tissue Sampling and Processing

Full-thickness skin specimens containing two of the six circular excisional wound lesions and the surrounding 3 mm uninjured skin area were obtained from 4 rats per study group for histological evaluation on the 2nd, 4th, 8th, and 15th days post-injury, after which the animals were sacrificed.

The excised tissues after being mounted on a piece of cork with push pins on each of the four corners—the deepest part of the dermis facing the cork—were fixed in 10% Formaldehyde solution for the first 24 h. The fixed tissue samples were then cut longitudinally, with the section–diameter line passed the deepest point/center of the excised wound, dividing the sample into two equal parts.

All specimens were then subjected to routine histological processing: step-by-step dehydration with ethanol, clearing with xylene, and wax infiltration with paraffin, to finally be embedded and oriented in paraffin blocks.

Each specimen was cut longitudinally into 3 μm thick sections: the 1st and 2nd slices, as well as the 5th and 6th, were processed and stained with Hematoxylin and Eosin staining (the 3rd and 4th sections were kept for immunostaining). Immunocytochemistry was performed with the use of Polyclonal Rabbit Anti-human CD117, c-kit by Dako. Thus, each wound—having been divided into two parts—was blindly assessed by two pathologists using two slices from each half, totaling eight sections per wound. For the proper assessment of neovascularization percentage, the immunohistochemical stain for smooth muscle actin (SMA) was performed. The specific antibody stains the smooth muscle cells and myofibrils in the vessel walls.

### 4.6. Cell Counting

The NIKON E200 Eclipse microscope was used for cell measurements in three consecutive high-power magnification fields (40×) per section. The mean and SD of these measurements were used for the final evaluation of the populations of cells involved in the healing process during the different time points of the study. The inflammatory cells, including neutrophils, lymphocytes, plasmacytes, mast cells, as well as fibroblasts, were individually counted by the two separate pathologists using the NIKON E200 Eclipse microscope, Nikon BioImaging Lab, Boston, MA, USA; Shonan, Japan. The cells were counted at the depth of the lesion in three consecutive high-power magnification fields (40×). The optical field, or field of view, has an area of 0.25 mm^2^, so the cell count refers to the number of cells per 0.25 mm^2^. In cases where granulation tissue was present at the top of the lesion, it was excluded from the measurements. The data were gathered and statistically evaluated according to the three predetermined groups.

For the study of neovascularization, tissue slices that were properly immunohistochemically stained were assessed. The percentage of newly formed capillary vessels within the depth of the tissue ulcer, specifically including the epidermal and dermal layers, was assessed. Preformed vessels or those with a muscular wall were not considered in the analysis. Using a microscope with a mm grid lens, the pathologist individually estimated the percentage of the newly formed capillary vessels within the ulcerated tissue depth. These percentages were then categorized into four histological groups. Group 0 denoted the absence of neovascularization, while Group 1 encompassed percentages ranging from 1 to 5%. Groups 2 and 3 represented vessel densities ranging between 6–20% and 21–39%, respectively. Notably, Group 4 exhibited the highest percentage of newly formed vessels, categorized as any percentage exceeding 40% of the complete surface of the ulcer depth.

### 4.7. Statistical Analysis

In order to express results, descriptive statistics were appropriately used. Means, medians, interquartile range, and standard deviations were used for continuous variables. The normal distribution of quantitative data has been checked using Kolmogorov–Smirnov test.

For the categorical variable, frequencies were calculated. 

Nonparametric tests (Mann–Whitney U test) were applied for the non-normal distribution data. Spearman rho test was applied for the correlation between continuous variables. Kruskall–Wallis test and Chi-square test were applied for the correlated data. Alpha was set at 0.05. Excel 2007 (Microsoft Office Excel 2007, Jones, Chicago, IL, USA) and SPSS 22.0 (IBM Corp. Released 2013. IBM SPSS Statistics for Windows, Version 22.0. Armonk, NY, USA: IBM Corp.) were employed to statistically analyze the data.

## 5. Conclusions

To conclude, the modulation of the wound microenvironment by beneficial bacteria, i.e., the probiotics, positively affects the healing process. As a result, wound healing is achieved earlier with the application of probiotic strains. Our study presents the cellular perspective of excisional wound healing, with diverse results among different healing phases and cellular counts. Probiotic regimens, with their anti-inflammatory and anti-fibrotic properties, prevent neutrophil and fibroblast exertion in the wound, while lymphocyte, mast cells, and plasmacyte counts are also divergent in comparison to the control group. Furthermore, both probiotic regimens enhanced neovascularization in the depth of the wound faster and better than the control group. Each strain, PRO1 and PRO2, works differently and more effectively in different healing phases, with PRO1 exhibiting better results earlier, while PRO2 showing a later onset but longer-lasting results. Finally, the broader application of probiotic regimens to the human population and assistance to skin wound healing is promoted. Thus, a combined formula containing different probiotics to modulate various healing phases is desirable.

## Figures and Tables

**Table 1:** Study groups and the number of rats used for healing assessment, per day of evaluation (two wounds per rat).

Study Groups	Day 2	Day 4	Day 8	Day 15
Control (Contr)	4	4	4	4
*L. plantarum* (PRO1)	4	4	4	4
*L. rhamnosus* and *B. longum* (PRO2)	4	4	4	4

## Data Availability

Data will be made available upon reasonable request.
